# Methyl 3-(1*H*-indole-3-carboxamido)propionate hemihydrate

**DOI:** 10.1107/S1600536810018015

**Published:** 2010-05-26

**Authors:** Gang Huang, Xing Yan Xu, Xiang Chao Zeng, Le Zheng, Kai Ping Li

**Affiliations:** a Department of Chemistry, Jinan University, Guangzhou, Guangdong 510632, People’s Republic of China

## Abstract

The title compound, C_13_H_14_N_2_O_3_·0.5H_2_O, was synthesized by the condensation of methyl 3-amino­propionate with 3-trichloro­acetyl­indole. The two organic mol­ecules in the asymmetric unit are both close to planar, with r.m.s. deviations from the best fit plane through all of the non-H atoms of 0.004 (2) Å for mol­ecule *A* and 0.006 (1) Å for mol­ecule *B*. Also, the five- and six-membered rings of the indole systems are inclined at 1.67 (8) and 1.50 (8)° in mol­ecules *A* and *B*, respectively. In the crystal structure, the organic molecules are connected by inter­molecular N—H⋯O hydrogen bonds, forming chains. O—H⋯O and N—H⋯O hydrogen-bond inter­actions involving the water molecules inter­link these chains, forming double chains approximately parallel to the *a* axis.

## Related literature

For the bioactivity of indole derivatives, see: Fabio *et al.* (2007 ▶); Sharma *et al.* (2004 ▶). For related structures, see: Huang *et al.* (2009 ▶); Siddiquee *et al.* (2009 ▶). For reference structural data, see Allen *et al.* (1987 ▶). 
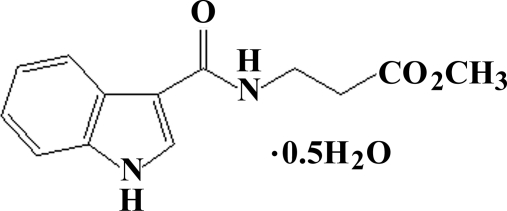


## Experimental

### 

#### Crystal data

C_13_H_14_N_2_O_3_·0.5H_2_O
*M* *_r_* = 255.27
Monoclinic, 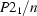

*a* = 10.8220 (13) Å
*b* = 9.7668 (12) Å
*c* = 23.623 (3) Å
β = 92.422 (2)°
*V* = 2494.6 (5) Å^3^
*Z* = 8
Mo *K*α radiation
μ = 0.10 mm^−1^
*T* = 173 K
0.46 × 0.43 × 0.39 mm

#### Data collection

Bruker SMART 1K CCD area-detector diffractometer
Absorption correction: multi-scan (*SADABS*; Sheldrick, 1996 ▶) *T*_min_ = 0.955, *T*_max_ = 0.962
13021 measured reflections
5484 independent reflections
4349 reflections with *I* > 2σ(*I*)
*R*_int_ = 0.036

#### Refinement

*R*[*F* ^2^ > 2σ(*F* ^2^)] = 0.042
*wR*(*F* ^2^) = 0.126
*S* = 1.07
5484 reflections
336 parameters
H-atom parameters constrained
Δρ_max_ = 0.29 e Å^−3^
Δρ_min_ = −0.24 e Å^−3^

### 

Data collection: *SMART* (Bruker, 1999 ▶); cell refinement: *SAINT-Plus* (Bruker, 1999 ▶); data reduction: *SAINT-Plus*; program(s) used to solve structure: *SHELXS97* (Sheldrick, 2008 ▶); program(s) used to refine structure: *SHELXL97* (Sheldrick, 2008 ▶); molecular graphics: *SHELXTL* (Sheldrick, 2008 ▶); software used to prepare material for publication: *SHELXTL*.

## Supplementary Material

Crystal structure: contains datablocks I, global. DOI: 10.1107/S1600536810018015/sj2794sup1.cif

Structure factors: contains datablocks I. DOI: 10.1107/S1600536810018015/sj2794Isup2.hkl

Additional supplementary materials:  crystallographic information; 3D view; checkCIF report

## Figures and Tables

**Table 1:** Hydrogen-bond geometry (Å, °)

*D*—H⋯*A*	*D*—H	H⋯*A*	*D*⋯*A*	*D*—H⋯*A*
N1—H1*A*⋯O4^i^	0.88	2.04	2.8219 (15)	148
N2—H2⋯O1*W*^ii^	0.88	2.09	2.9348 (17)	161
N3—H3⋯O1	0.88	1.88	2.7404 (16)	164
N4—H4*A*⋯O2	0.88	2.43	3.2952 (15)	167
O1*W*—H1*B*⋯O4	0.85	2.17	2.9890 (18)	161
O1*W*—H1*C*⋯O5^iii^	0.85	1.98	2.7970 (16)	160

Symmetry codes: (i) 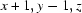; (ii) 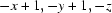; (iii) 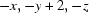.

**Fig. 1.**
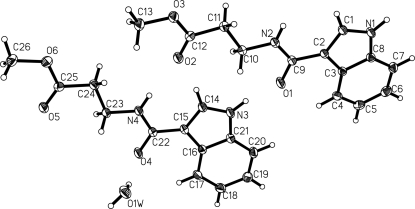
The asymmetric unit of the title compound, with the atom-numbering scheme. Displacement ellipsoids are drawn at the 30% probability level.

**Fig. 2.**
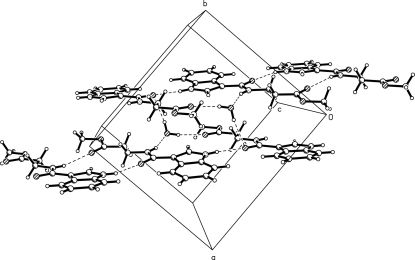
Crystal packing of the hydrate (viewed along the c axis). Dashed lines indicate hydrogen bonds.

## supplementary crystallographic information

### Comment

Many indole derivatives show important bioactivity, acting as metabotropic receptor antagonists (Fabio *et al.*, 2007) and showing protein kinase inhibiting activity (Sharma *et al.*, 2004). This is the reason they have attracted our interest and the title compound is related to our previous structural investigations of methyl 3-(1-Butyl-1*H*-indole-3-carbonyl)aminopropionate (Huang *et al.*, 2009).

The asymmetric unit of the title compound comprises two substituted indole molecules, A & B and a solvent water molecule, Fig. 1. The organic molecules are each reasonably planar with rms deviations from the best fit plane through all of the non hydrogen atoms of 0.004 (2) Å for A and -0.006 (1) Å for B. Also the five and six-membered rings of the indole systems are inclined at 1.67 (8) ° and 1.50 (8) ° in A and B respectively. Bond lengths in both molecules are unexceptional (Allen *et al.*, 1987) and are comparable to those found in similar structures (Huang *et al.*, 2009; Siddiquee *et al.*, 2009).

In the crystal structure, molecules of the organic compound are linked through N—H···O H-bonds to form dimers that are connected by another N—H···O H-bonds to generate chains, and the O—H(W)···O and N—H···O(W) H-bond interactions link these chains to form double-chains extending to the *a* axis (Table 1, Fig. 2).

### Experimental

The hydrochloride salt of methyl 3-aminopropionate (0.70 g, 5 mmol) and 3-trichloroacetylindole (1.32 g, 5 mmol) were added to acetonitrile (10 ml), followed by the dropwise addition of triethylamine (1.2 ml). The mixture was stirred at room temperature for 16 h and then poured into water. After filtration, the precipitate was collected as a yellow solid. The impure product was dissolved in EtOH at room temperature. Light yellow monoclinic crystals suitable for X-ray analysis (m.p. 409 K, 86.2% yield) grew over a period of one week when the solution was exposed to the air.

### Refinement

All non-H atoms were refined with anisotropic displacement parameters. The H atoms bound to C and N were positioned geometrically [C—H = 0.99Å for CH_2_, 0.98Å for CH_3_, 0.95Å for CH(aromatic) and N—H = 0.88 Å] and refined using a riding model, with *U*_iso_ = 1.2*U*_eq_ (1.5*U*_eq_ for the methyl group) of the parent atom. The water H atoms were located in a difference Fourier map and were constrained in these positions with  O—H = 0.8497 and 0.8533, and with U_iso_ = 1.5U_eq_(O).

### Crystal data

**Table:**

C_13_H_14_N_2_O_3_·0.5H_2_O	*F*(000) = 1080
*M**_r_* = 255.27	*D*_x_ = 1.359 Mg m^−^^3^
Monoclinic, *P*2_1_/*n*	Melting point: 409 K
Hall symbol: -P 2yn	Mo *K*α radiation, λ = 0.71073 Å
*a* = 10.8220 (13) Å	Cell parameters from 7482 reflections
*b* = 9.7668 (12) Å	θ = 2.3–27.1°
*c* = 23.623 (3) Å	µ = 0.10 mm^−^^1^
β = 92.422 (2)°	*T* = 173 K
*V* = 2494.6 (5) Å^3^	Block, yellow
*Z* = 8	0.46 × 0.43 × 0.39 mm

### Data collection

**Table:**

Bruker SMART 1K CCD area-detector diffractometer	5484 independent reflections
Radiation source: fine-focus sealed tube	4349 reflections with *I* > 2σ(*I*)
graphite	*R*_int_ = 0.036
φ and ω scans	θ_max_ = 27.1°, θ_min_ = 1.7°
Absorption correction: multi-scan (*SADABS*; Sheldrick, 1996)	*h* = −13→11
*T*_min_ = 0.955, *T*_max_ = 0.962	*k* = −8→12
13021 measured reflections	*l* = −30→30

### Refinement

**Table:**

Refinement on *F*^2^	Primary atom site location: structure-invariant direct methods
Least-squares matrix: full	Secondary atom site location: difference Fourier map
*R*[*F*^2^ > 2σ(*F*^2^)] = 0.042	Hydrogen site location: inferred from neighbouring sites
*wR*(*F*^2^) = 0.126	H-atom parameters constrained
*S* = 1.07	*w* = 1/[σ^2^(*F*_o_^2^) + (0.0626*P*)^2^ + 0.3718*P*] where *P* = (*F*_o_^2^ + 2*F*_c_^2^)/3
5484 reflections	(Δ/σ)_max_ = 0.001
336 parameters	Δρ_max_ = 0.28 e Å^−^^3^
0 restraints	Δρ_min_ = −0.23 e Å^−^^3^

### Special details

**Table:**

Geometry. All esds (except the esd in the dihedral angle between two l.s. planes) are estimated using the full covariance matrix. The cell esds are taken into account individually in the estimation of esds in distances, angles and torsion angles; correlations between esds in cell parameters are only used when they are defined by crystal symmetry. An approximate (isotropic) treatment of cell esds is used for estimating esds involving l.s. planes.
Refinement. Refinement of *F*^2^ against ALL reflections. The weighted *R*-factor wR and goodness of fit *S* are based on *F*^2^, conventional *R*-factors *R* are based on *F*, with *F* set to zero for negative *F*^2^. The threshold expression of *F*^2^ > σ(*F*^2^) is used only for calculating *R*-factors(gt) etc. and is not relevant to the choice of reflections for refinement. *R*-factors based on *F*^2^ are statistically about twice as large as those based on *F*, and *R*- factors based on ALL data will be even larger.

### Fractional atomic coordinates and isotropic or equivalent isotropic displacement parameters (Å^2^)

**Table:**

	*x*	*y*	*z*	*U*_iso_*/*U*_eq_	
C1	0.80813 (13)	−0.08599 (15)	0.03833 (6)	0.0318 (3)	
H1	0.8269	−0.0666	0.0002	0.038*	
C2	0.71838 (12)	−0.02181 (14)	0.06810 (6)	0.0272 (3)	
C3	0.72133 (12)	−0.08192 (14)	0.12390 (6)	0.0275 (3)	
C4	0.65431 (14)	−0.06387 (16)	0.17297 (6)	0.0341 (3)	
H4	0.5917	0.0041	0.1743	0.041*	
C5	0.68093 (15)	−0.14651 (17)	0.21922 (6)	0.0398 (4)	
H5	0.6352	−0.1356	0.2524	0.048*	
C6	0.77349 (16)	−0.24579 (17)	0.21840 (7)	0.0421 (4)	
H6	0.7892	−0.3012	0.2510	0.051*	
C7	0.84253 (14)	−0.26528 (16)	0.17145 (7)	0.0373 (3)	
H7	0.9061	−0.3323	0.1710	0.045*	
C8	0.81497 (12)	−0.18210 (14)	0.12439 (6)	0.0297 (3)	
C9	0.62911 (13)	0.08453 (15)	0.04984 (6)	0.0305 (3)	
C10	0.53881 (13)	0.24140 (15)	−0.01892 (6)	0.0311 (3)	
H10A	0.5536	0.3226	0.0055	0.037*	
H10B	0.4544	0.2074	−0.0125	0.037*	
C11	0.54663 (13)	0.28339 (15)	−0.08026 (6)	0.0321 (3)	
H11A	0.6316	0.3150	−0.0872	0.039*	
H11B	0.5286	0.2034	−0.1050	0.039*	
C12	0.45597 (13)	0.39641 (15)	−0.09478 (6)	0.0308 (3)	
C13	0.39062 (16)	0.55673 (18)	−0.16381 (7)	0.0433 (4)	
H13A	0.3959	0.6307	−0.1358	0.065*	
H13B	0.3056	0.5222	−0.1670	0.065*	
H13C	0.4145	0.5917	−0.2007	0.065*	
C14	0.29377 (13)	0.36840 (16)	0.06182 (6)	0.0341 (3)	
H14	0.3102	0.3692	0.0226	0.041*	
C15	0.21024 (12)	0.45232 (14)	0.08725 (6)	0.0290 (3)	
C16	0.21441 (12)	0.41712 (14)	0.14649 (6)	0.0284 (3)	
C17	0.15418 (14)	0.46235 (16)	0.19450 (6)	0.0354 (3)	
H17	0.0928	0.5317	0.1913	0.042*	
C18	0.18574 (16)	0.40417 (17)	0.24654 (7)	0.0432 (4)	
H18	0.1446	0.4335	0.2791	0.052*	
C19	0.27677 (16)	0.30334 (18)	0.25209 (7)	0.0441 (4)	
H19	0.2977	0.2669	0.2885	0.053*	
C20	0.33663 (15)	0.25580 (17)	0.20592 (7)	0.0399 (4)	
H20	0.3981	0.1866	0.2097	0.048*	
C21	0.30395 (13)	0.31267 (15)	0.15322 (6)	0.0319 (3)	
C22	0.13786 (12)	0.56060 (14)	0.05926 (6)	0.0290 (3)	
C23	0.08226 (13)	0.69102 (15)	−0.02576 (6)	0.0301 (3)	
H23A	0.1048	0.7807	−0.0088	0.036*	
H23B	−0.0072	0.6763	−0.0211	0.036*	
C24	0.10799 (13)	0.69297 (15)	−0.08814 (6)	0.0328 (3)	
H24A	0.1972	0.7095	−0.0929	0.039*	
H24B	0.0866	0.6029	−0.1051	0.039*	
C25	0.03353 (13)	0.80346 (15)	−0.11816 (6)	0.0310 (3)	
C26	−0.01206 (18)	0.91058 (19)	−0.20573 (7)	0.0497 (4)	
H26A	−0.1005	0.8992	−0.1997	0.075*	
H26B	0.0033	0.8978	−0.2460	0.075*	
H26C	0.0139	1.0028	−0.1940	0.075*	
N1	0.86589 (11)	−0.18166 (13)	0.07191 (5)	0.0333 (3)	
H1A	0.9265	−0.2351	0.0617	0.040*	
N2	0.62849 (11)	0.13484 (13)	−0.00271 (5)	0.0317 (3)	
H2	0.6811	0.1044	−0.0272	0.038*	
N3	0.34897 (12)	0.28454 (14)	0.10095 (5)	0.0375 (3)	
H3	0.4048	0.2220	0.0940	0.045*	
N4	0.15203 (11)	0.58337 (12)	0.00368 (5)	0.0320 (3)	
H4A	0.2039	0.5327	−0.0150	0.038*	
O1	0.55464 (11)	0.12826 (13)	0.08443 (5)	0.0466 (3)	
O2	0.37724 (10)	0.43840 (12)	−0.06434 (4)	0.0405 (3)	
O3	0.47321 (10)	0.44687 (12)	−0.14607 (4)	0.0393 (3)	
O4	0.06489 (10)	0.63299 (11)	0.08581 (4)	0.0396 (3)	
O5	−0.03955 (10)	0.87601 (12)	−0.09532 (4)	0.0402 (3)	
O6	0.05767 (10)	0.80986 (12)	−0.17246 (4)	0.0413 (3)	
O1W	0.20002 (11)	0.89955 (13)	0.09463 (5)	0.0507 (3)	
H1B	0.1537	0.8301	0.0994	0.076*	
H1C	0.1592	0.9704	0.1031	0.076*	

### Atomic displacement parameters (Å^2^)

**Table:**

	*U*^11^	*U*^22^	*U*^33^	*U*^12^	*U*^13^	*U*^23^
C1	0.0314 (7)	0.0312 (7)	0.0330 (7)	0.0062 (6)	0.0043 (5)	0.0008 (6)
C2	0.0245 (6)	0.0256 (6)	0.0314 (7)	0.0024 (5)	0.0014 (5)	−0.0018 (5)
C3	0.0257 (6)	0.0249 (6)	0.0317 (7)	0.0006 (5)	−0.0005 (5)	−0.0032 (5)
C4	0.0347 (7)	0.0341 (8)	0.0338 (7)	0.0017 (6)	0.0035 (6)	−0.0060 (6)
C5	0.0477 (9)	0.0418 (9)	0.0303 (7)	−0.0043 (7)	0.0050 (6)	−0.0012 (6)
C6	0.0537 (10)	0.0372 (8)	0.0349 (8)	−0.0022 (7)	−0.0066 (7)	0.0058 (7)
C7	0.0395 (8)	0.0307 (7)	0.0411 (8)	0.0050 (6)	−0.0056 (6)	0.0029 (6)
C8	0.0279 (7)	0.0270 (7)	0.0341 (7)	0.0006 (5)	−0.0005 (5)	−0.0024 (6)
C9	0.0288 (7)	0.0297 (7)	0.0331 (7)	0.0048 (6)	−0.0001 (5)	−0.0024 (6)
C10	0.0319 (7)	0.0298 (7)	0.0315 (7)	0.0067 (6)	−0.0002 (5)	−0.0002 (6)
C11	0.0298 (7)	0.0335 (7)	0.0329 (7)	0.0029 (6)	0.0000 (6)	−0.0017 (6)
C12	0.0311 (7)	0.0322 (7)	0.0289 (7)	−0.0009 (6)	−0.0006 (5)	−0.0002 (6)
C13	0.0491 (9)	0.0457 (9)	0.0353 (8)	0.0135 (8)	0.0020 (7)	0.0086 (7)
C14	0.0337 (7)	0.0351 (8)	0.0337 (7)	0.0100 (6)	0.0053 (6)	0.0028 (6)
C15	0.0259 (6)	0.0268 (7)	0.0345 (7)	0.0041 (5)	0.0027 (5)	0.0002 (6)
C16	0.0268 (6)	0.0243 (6)	0.0341 (7)	0.0008 (5)	0.0004 (5)	0.0001 (5)
C17	0.0389 (8)	0.0294 (7)	0.0381 (8)	0.0072 (6)	0.0045 (6)	−0.0022 (6)
C18	0.0561 (10)	0.0393 (9)	0.0347 (8)	0.0068 (7)	0.0078 (7)	−0.0024 (7)
C19	0.0588 (10)	0.0387 (9)	0.0344 (8)	0.0063 (8)	−0.0015 (7)	0.0052 (7)
C20	0.0431 (8)	0.0356 (8)	0.0409 (8)	0.0106 (7)	−0.0001 (7)	0.0071 (7)
C21	0.0304 (7)	0.0303 (7)	0.0352 (7)	0.0044 (6)	0.0033 (6)	0.0009 (6)
C22	0.0263 (6)	0.0261 (7)	0.0348 (7)	0.0031 (5)	0.0018 (5)	0.0007 (6)
C23	0.0299 (7)	0.0281 (7)	0.0324 (7)	0.0049 (6)	0.0010 (5)	0.0012 (6)
C24	0.0331 (7)	0.0315 (7)	0.0341 (7)	0.0051 (6)	0.0048 (6)	−0.0007 (6)
C25	0.0335 (7)	0.0303 (7)	0.0293 (7)	−0.0022 (6)	0.0032 (5)	−0.0012 (6)
C26	0.0705 (12)	0.0463 (10)	0.0321 (8)	0.0024 (9)	−0.0013 (8)	0.0089 (7)
N1	0.0307 (6)	0.0316 (6)	0.0378 (7)	0.0109 (5)	0.0052 (5)	0.0001 (5)
N2	0.0305 (6)	0.0324 (6)	0.0323 (6)	0.0086 (5)	0.0027 (5)	0.0000 (5)
N3	0.0371 (7)	0.0375 (7)	0.0381 (7)	0.0170 (6)	0.0061 (5)	0.0035 (5)
N4	0.0322 (6)	0.0307 (6)	0.0333 (6)	0.0102 (5)	0.0046 (5)	0.0017 (5)
O1	0.0473 (6)	0.0551 (7)	0.0382 (6)	0.0289 (6)	0.0098 (5)	0.0055 (5)
O2	0.0419 (6)	0.0452 (6)	0.0348 (5)	0.0147 (5)	0.0070 (4)	0.0054 (5)
O3	0.0441 (6)	0.0426 (6)	0.0314 (5)	0.0128 (5)	0.0050 (4)	0.0057 (5)
O4	0.0419 (6)	0.0392 (6)	0.0381 (6)	0.0197 (5)	0.0084 (4)	0.0045 (5)
O5	0.0467 (6)	0.0393 (6)	0.0350 (5)	0.0148 (5)	0.0056 (5)	0.0025 (5)
O6	0.0523 (7)	0.0422 (6)	0.0298 (5)	0.0042 (5)	0.0061 (5)	0.0037 (5)
O1W	0.0556 (7)	0.0471 (7)	0.0506 (7)	0.0176 (6)	0.0152 (6)	0.0011 (6)

### Geometric parameters (Å, °)

**Table:**

C1—N1	1.3606 (18)	C15—C16	1.4399 (19)
C1—C2	1.3744 (19)	C15—C22	1.4579 (19)
C1—H1	0.9500	C16—C17	1.403 (2)
C2—C3	1.4421 (19)	C16—C21	1.4114 (19)
C2—C9	1.4706 (19)	C17—C18	1.384 (2)
C3—C4	1.404 (2)	C17—H17	0.9500
C3—C8	1.4083 (19)	C18—C19	1.395 (2)
C4—C5	1.379 (2)	C18—H18	0.9500
C4—H4	0.9500	C19—C20	1.373 (2)
C5—C6	1.395 (2)	C19—H19	0.9500
C5—H5	0.9500	C20—C21	1.395 (2)
C6—C7	1.376 (2)	C20—H20	0.9500
C6—H6	0.9500	C21—N3	1.3743 (19)
C7—C8	1.399 (2)	C22—O4	1.2485 (17)
C7—H7	0.9500	C22—N4	1.3467 (18)
C8—N1	1.3779 (19)	C23—N4	1.4543 (17)
C9—O1	1.2470 (17)	C23—C24	1.511 (2)
C9—N2	1.3348 (18)	C23—H23A	0.9900
C10—N2	1.4630 (17)	C23—H23B	0.9900
C10—C11	1.512 (2)	C24—C25	1.506 (2)
C10—H10A	0.9900	C24—H24A	0.9900
C10—H10B	0.9900	C24—H24B	0.9900
C11—C12	1.507 (2)	C25—O5	1.2062 (18)
C11—H11A	0.9900	C25—O6	1.3212 (17)
C11—H11B	0.9900	C26—O6	1.451 (2)
C12—O2	1.2095 (17)	C26—H26A	0.9800
C12—O3	1.3285 (17)	C26—H26B	0.9800
C13—O3	1.4470 (19)	C26—H26C	0.9800
C13—H13A	0.9800	N1—H1A	0.8800
C13—H13B	0.9800	N2—H2	0.8800
C13—H13C	0.9800	N3—H3	0.8800
C14—N3	1.3548 (19)	N4—H4A	0.8800
C14—C15	1.377 (2)	O1W—H1B	0.8533
C14—H14	0.9500	O1W—H1C	0.8497
			
N1—C1—C2	109.39 (12)	C21—C16—C15	105.98 (12)
N1—C1—H1	125.3	C18—C17—C16	118.89 (14)
C2—C1—H1	125.3	C18—C17—H17	120.6
C1—C2—C3	107.05 (12)	C16—C17—H17	120.6
C1—C2—C9	129.92 (13)	C17—C18—C19	121.33 (15)
C3—C2—C9	122.99 (12)	C17—C18—H18	119.3
C4—C3—C8	118.49 (13)	C19—C18—H18	119.3
C4—C3—C2	135.33 (13)	C20—C19—C18	121.28 (15)
C8—C3—C2	106.17 (12)	C20—C19—H19	119.4
C5—C4—C3	118.86 (14)	C18—C19—H19	119.4
C5—C4—H4	120.6	C19—C20—C21	117.64 (14)
C3—C4—H4	120.6	C19—C20—H20	121.2
C4—C5—C6	121.47 (15)	C21—C20—H20	121.2
C4—C5—H5	119.3	N3—C21—C20	129.50 (13)
C6—C5—H5	119.3	N3—C21—C16	108.12 (12)
C7—C6—C5	121.46 (15)	C20—C21—C16	122.37 (14)
C7—C6—H6	119.3	O4—C22—N4	119.79 (12)
C5—C6—H6	119.3	O4—C22—C15	121.47 (13)
C6—C7—C8	117.08 (14)	N4—C22—C15	118.73 (12)
C6—C7—H7	121.5	N4—C23—C24	111.22 (11)
C8—C7—H7	121.5	N4—C23—H23A	109.4
N1—C8—C7	129.56 (13)	C24—C23—H23A	109.4
N1—C8—C3	107.79 (12)	N4—C23—H23B	109.4
C7—C8—C3	122.63 (14)	C24—C23—H23B	109.4
O1—C9—N2	120.34 (13)	H23A—C23—H23B	108.0
O1—C9—C2	118.97 (13)	C25—C24—C23	110.56 (11)
N2—C9—C2	120.68 (12)	C25—C24—H24A	109.5
N2—C10—C11	112.37 (11)	C23—C24—H24A	109.5
N2—C10—H10A	109.1	C25—C24—H24B	109.5
C11—C10—H10A	109.1	C23—C24—H24B	109.5
N2—C10—H10B	109.1	H24A—C24—H24B	108.1
C11—C10—H10B	109.1	O5—C25—O6	124.53 (13)
H10A—C10—H10B	107.9	O5—C25—C24	123.92 (13)
C12—C11—C10	110.78 (12)	O6—C25—C24	111.54 (12)
C12—C11—H11A	109.5	O6—C26—H26A	109.5
C10—C11—H11A	109.5	O6—C26—H26B	109.5
C12—C11—H11B	109.5	H26A—C26—H26B	109.5
C10—C11—H11B	109.5	O6—C26—H26C	109.5
H11A—C11—H11B	108.1	H26A—C26—H26C	109.5
O2—C12—O3	123.10 (13)	H26B—C26—H26C	109.5
O2—C12—C11	125.54 (13)	C1—N1—C8	109.60 (11)
O3—C12—C11	111.36 (12)	C1—N1—H1A	125.2
O3—C13—H13A	109.5	C8—N1—H1A	125.2
O3—C13—H13B	109.5	C9—N2—C10	118.86 (11)
H13A—C13—H13B	109.5	C9—N2—H2	120.6
O3—C13—H13C	109.5	C10—N2—H2	120.6
H13A—C13—H13C	109.5	C14—N3—C21	109.23 (12)
H13B—C13—H13C	109.5	C14—N3—H3	125.4
N3—C14—C15	109.93 (13)	C21—N3—H3	125.4
N3—C14—H14	125.0	C22—N4—C23	120.49 (11)
C15—C14—H14	125.0	C22—N4—H4A	119.8
C14—C15—C16	106.73 (12)	C23—N4—H4A	119.8
C14—C15—C22	125.74 (13)	C12—O3—C13	115.49 (12)
C16—C15—C22	127.45 (12)	C25—O6—C26	116.00 (12)
C17—C16—C21	118.46 (13)	H1B—O1W—H1C	107.6
C17—C16—C15	135.56 (13)		
			
N1—C1—C2—C3	0.15 (16)	C16—C17—C18—C19	0.8 (3)
N1—C1—C2—C9	−177.67 (14)	C17—C18—C19—C20	−1.5 (3)
C1—C2—C3—C4	−179.04 (15)	C18—C19—C20—C21	0.5 (3)
C9—C2—C3—C4	−1.0 (2)	C19—C20—C21—N3	179.75 (16)
C1—C2—C3—C8	−0.29 (15)	C19—C20—C21—C16	1.1 (2)
C9—C2—C3—C8	177.72 (12)	C17—C16—C21—N3	179.37 (13)
C8—C3—C4—C5	−1.3 (2)	C15—C16—C21—N3	−1.26 (16)
C2—C3—C4—C5	177.35 (15)	C17—C16—C21—C20	−1.7 (2)
C3—C4—C5—C6	0.8 (2)	C15—C16—C21—C20	177.67 (14)
C4—C5—C6—C7	0.2 (2)	C14—C15—C22—O4	−179.26 (14)
C5—C6—C7—C8	−0.6 (2)	C16—C15—C22—O4	−3.1 (2)
C6—C7—C8—N1	−177.99 (14)	C14—C15—C22—N4	−0.2 (2)
C6—C7—C8—C3	0.1 (2)	C16—C15—C22—N4	175.97 (13)
C4—C3—C8—N1	179.32 (12)	N4—C23—C24—C25	179.09 (12)
C2—C3—C8—N1	0.31 (15)	C23—C24—C25—O5	−2.6 (2)
C4—C3—C8—C7	0.9 (2)	C23—C24—C25—O6	177.96 (12)
C2—C3—C8—C7	−178.12 (13)	C2—C1—N1—C8	0.05 (16)
C1—C2—C9—O1	179.73 (15)	C7—C8—N1—C1	178.05 (15)
C3—C2—C9—O1	2.2 (2)	C3—C8—N1—C1	−0.23 (16)
C1—C2—C9—N2	−1.0 (2)	O1—C9—N2—C10	−0.2 (2)
C3—C2—C9—N2	−178.56 (12)	C2—C9—N2—C10	−179.46 (12)
N2—C10—C11—C12	−178.09 (12)	C11—C10—N2—C9	−177.74 (12)
C10—C11—C12—O2	−8.0 (2)	C15—C14—N3—C21	−0.72 (18)
C10—C11—C12—O3	171.58 (12)	C20—C21—N3—C14	−177.58 (16)
N3—C14—C15—C16	−0.08 (17)	C16—C21—N3—C14	1.25 (17)
N3—C14—C15—C22	176.72 (13)	O4—C22—N4—C23	−0.5 (2)
C14—C15—C16—C17	−179.97 (16)	C15—C22—N4—C23	−179.56 (13)
C22—C15—C16—C17	3.3 (3)	C24—C23—N4—C22	−177.04 (12)
C14—C15—C16—C21	0.82 (16)	O2—C12—O3—C13	−0.2 (2)
C22—C15—C16—C21	−175.91 (14)	C11—C12—O3—C13	−179.74 (13)
C21—C16—C17—C18	0.7 (2)	O5—C25—O6—C26	−1.2 (2)
C15—C16—C17—C18	−178.40 (16)	C24—C25—O6—C26	178.26 (13)

### Hydrogen-bond geometry (Å, °)

**Table:**

*D*—H···*A*	*D*—H	H···*A*	*D*···*A*	*D*—H···*A*
N1—H1A···O4^i^	0.88	2.04	2.8219 (15)	148
N2—H2···O1W^ii^	0.88	2.09	2.9348 (17)	161
N3—H3···O1	0.88	1.88	2.7404 (16)	164
N4—H4A···O2	0.88	2.43	3.2952 (15)	167
O1W—H1B···O4	0.85	2.17	2.9890 (18)	161
O1W—H1C···O5^iii^	0.85	1.98	2.7970 (16)	160

Symmetry codes: (i) *x*+1, *y*−1, *z*; (ii) −*x*+1, −*y*+1, −*z*; (iii) −*x*, −*y*+2, −*z*.
